# *Solanum incanum* extract (SR-T100) induces melanoma cell apoptosis and inhibits established lung metastasis

**DOI:** 10.18632/oncotarget.21508

**Published:** 2017-10-04

**Authors:** Sebastian Yu, Hamm-Ming Sheu, Chih-Hung Lee

**Affiliations:** ^1^ Graduate Institute of Clinical Medicine, College of Medicine, Kaohsiung Medical University, Kaohsiung, Taiwan; ^2^ Department of Dermatology, Kaohsiung Medical University Hospital, Kaohsiung Medical University, Kaohsiung, Taiwan; ^3^ Department of Dermatology, National Cheng Kung University College of Medicine and Hospital, Tainan, Taiwan; ^4^ Department of Dermatology, Kaohsiung Chang Gung Memorial Hospital, Chang Gung University College of Medicine, Kaohsiung, Taiwan

**Keywords:** melanoma, SR-T100, solamargine, Solanum incanum, immunotherapy

## Abstract

Melanoma, a cancer derived from melanocytes, is one of the most chemoresistant cancers and tends to metastasize. Once it metastasizes, the prognosis is poor. Even with the recent advancement of targeted therapy and immunotherapy, the prognosis remains discouraging. SR-T100, a *Solanum incanum* extract, shows anticancer effects against several cancers; however, its therapeutic efficacy against melanoma and established metastasis remains unknown. In this study, we showed that SR-T100 induces apoptosis, DNA damage, and G0/G1 cell cycle arrest in murine B16 melanoma cells *in vitro*. *In vivo*, intralesional injection of SR-T100 decreased the tumor size of the regional melanoma in the foot pad. Moreover, intraperitoneal injection of SR-T100 inhibited the growth and the number of established melanoma metastases in the lungs. Our study highlights SR-T100 as a potential novel treatment for established tumors from regional and metastatic melanoma.

## INTRODUCTION

Melanoma is one of the most debilitating human cancers. The annual productivity loss attributed to melanoma mortality was estimated to be $3.5 billion in the United States [1]. Although less common than other skin cancers such as squamous cell carcinoma (SCC) and basal cell carcinoma (BCC), melanoma accounts for the majority of skin cancer-related mortality due to its high propensity to metastasize. Once melanoma metastasizes, the prognosis is poor. The one-year survival rate of patients with stage IV metastatic melanoma is only 41–59% [2]. Even with the advance of targeted therapy, the overall median survival of metastatic melanoma is only 13.6 months [3]. More recently, combined immunotherapy with concurrent cytotoxic T-lymphocyte–associated antigen 4 (CTLA-4) and programmed death 1 (PD-1) blockade showed better progression-free survival than anti-CTLA-4 monotherapy [4]. However, severe drug-related adverse effects were reported in 54% of patients who received the combination immunotherapy. Emerging therapeutics for metastatic melanoma are needed to improve survival.

*Solanum* species extracts have been documented as clinical regimens for anticancer therapy for centuries [5, 6]. Previous reports showed that *Solanum* species extracts induce anticancer effects against human hepatoma cells [7], human lung cancer cells [8], human breast cancer cells [9–11], and human ovarian cancer cells [12] *in vitro*. Glycoalkaloids, including solamargine, solasodine, and solasonine, are active components, which are responsible for the anticancer activity of *Solanum* species [5, 6, 13]. More specifically, solamargine induces apoptosis of cancer cells by up-regulating Fas and tumor necrosis factor receptors [14–16] and by activating the mitochondrial apoptotic pathway [8, 9]. SR-T100, an extract from *Solanum incanum*, contains solamargine as its main active ingredient. SR-T100 induces apoptosis of human SCC cells both *in vitro* and *in vivo* [17]. Animal experiments showed that topical SR-T100 application resolved ultraviolet B-induced SCC in mice [17]. A small clinical trial showed that 10 out of 14 actinic keratosis lesions were cured after once-daily application of SR-T100 gel for 16 weeks [17]. Although SR-T100 is effective for treating SCC, the efficacy of SR-T100 has not been established for melanoma and related metastasis. In the present study, we investigated the therapeutic effect and the potential mechanisms of SR-T100 against metastatic melanoma *in vitro* and *in vivo*.

## RESULTS

### Cell viability and cell cycle arrest

**Figure 1:**
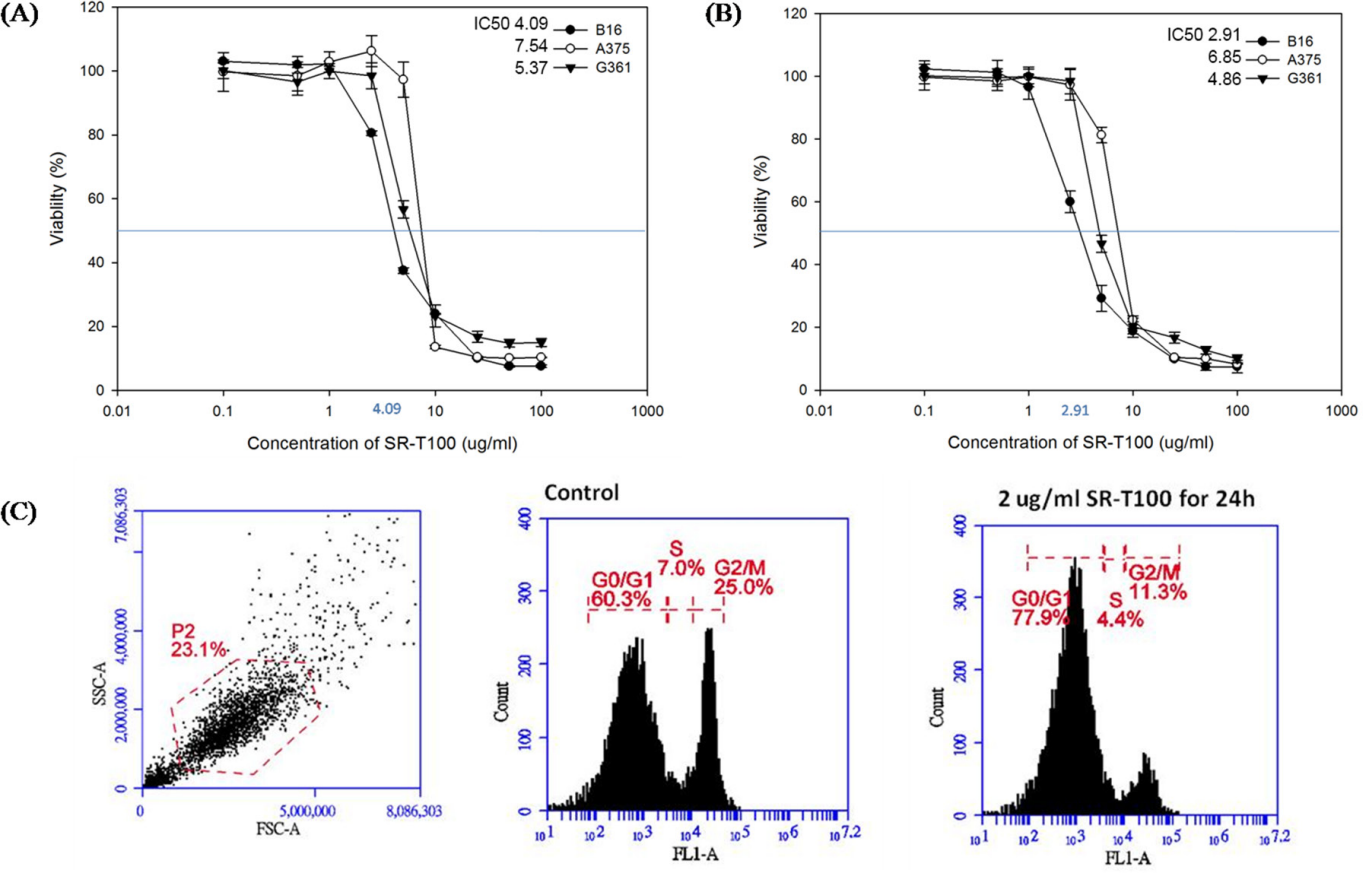
Effects of SR-T100 on the viability of B16, A375, and G361 cells at 24 h **(A)** and 48 h **(B)**. The IC50 (half maximal inhibitory concentration) values for SR-T100 on B16, A375, and G361 cells are shown (n = 3 for each time point, error bars indicated standard deviations). **(C)** SR-T100 induced G0/G1 cell cycle arrest. B16 cells were treated with 2 μg/mL of SR-T100 for 24 h and stained with Propidium Iodide (PI). Cells were analyzed with the FACScan flow cytometer. The percentage of cell counts of each cell cycle phase in a representative experiment is shown (G0/G1, S, G2/M) (n = 3).

To investigate whether SR-T100 affected cell viability, we treated B16 cells, derived from murine melanoma, along with A375 cells and G361 cells, both of which derived from human melanoma, with SR-T100 and measured cell viability by cytotoxicity assay. The results showed that SR-T100 has a dose-dependent killing effect on B16, A475, and G361 cells (Figure 1A). Based on the dose response curves, the half maximal inhibitory concentration (IC50) of SR-T100 at 24 hours towards B16, A375, and G361 cells was 4.09, 7.54, and 5.37 μg/mL, respectively. In addition, after 48 h treatment, the SR-T100 concentration required to induce the death of 50% of B16, A375, and G361 cells was 2.91, 6.85, and 4.86 μg/mL, respectively (Figure 1B). To further determine whether SR-T100 affected cell cycle progression, the proportion of SR-T100-treated B16 cells in each cell cycle phase was analyzed by flow cytometry (Figure 1C). While the percentage of cells in G0/G1 phase, S phase, and G2/M phase in non-treated B16 cells were 59.3 ± 3.4, 7.2 ± 2.1, and 24.6 ±4.3%, respectively (n = 3, mean ± S.D.), the percentage of B16 cells treated with 2 μg/mL SR-T100 in G0/G1 phase, S phase, and G2/M phase were 76.3 ± 7.4, 4.2 ± 1.6, 11.5 ± 3.8 %, respectively (n = 3, mean ± S.D.). There was a significant G0/G1 arrest in cells treated with SR-T100 (p < 0.05).

### Increased cell apoptosis and enhanced DNA damage in SR-T100-treated B16 cells

**Figure 2:**
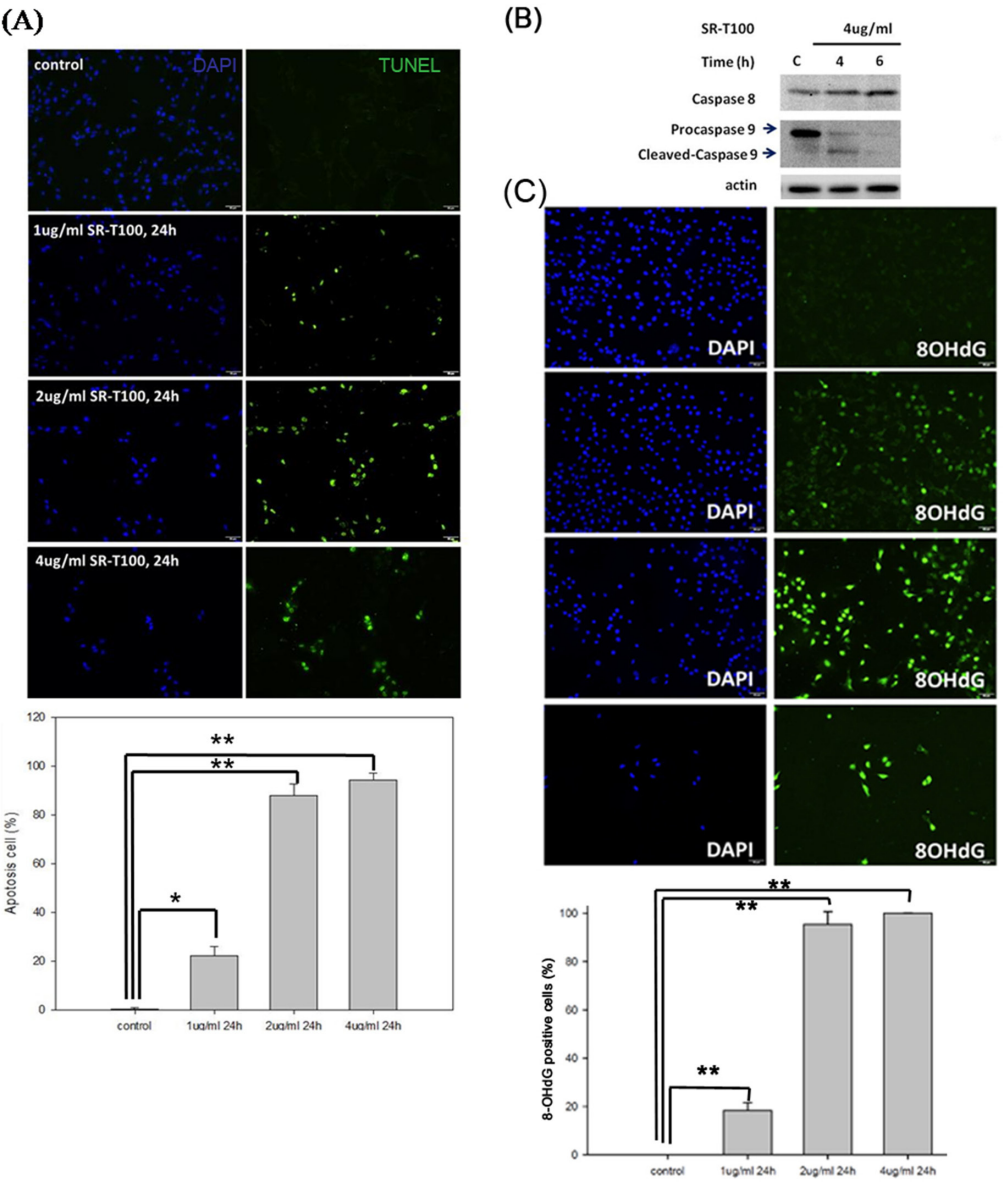
SR-T100 induces apoptosis and DNA damage in melanoma cell lines **(A)** To measure apoptosis, percentage of TUNEL positive cells was defined as TUNEL-positive cells among DAPI-positive cells (Experiments were repeated three times in triplicate). **(B)** Caspase 9 activity was measured by the cleavage of procaspase by western blot. (n = 3, a representative blot is shown). **(C)** To assess DNA damage, cells were treated with SR-T100 and stained for 8-OHdG and DAPI by immunofluorescence. Experiments were repeated three times in triplicate. ^*^ indicates p < 0.05, ^**^ indicates p < 0.01.

To evaluate whether SR-T100 induced apoptosis in B16 cells, the terminal deoxynucleotidyl transferase dUTP nick end labeling (TUNEL) assay was performed (Figure 2A). The results showed that SR-T100 induced apoptosis in B16 cells in a dose-dependent manner. Significant apoptosis (>80%) was induced by SR-T100 at 2 μg/mL for 24 hours. To further confirm whether apoptosis was induced by SR-T100, we performed western blot to measure the expression and activation of caspases. The results showed that cleavage of procaspase 9 was increased, while caspase 8 expression was not changed in B16 cells treated with 4 μg/mL SR-T100 (Figure 2B). Next, we investigated whether SR-T100 induced DNA damage. Using the same treatment protocol, 8-hydroxy-2’-deoxyguanosine (8-OHdG) assay was performed to examine DNA damage induced by SR-T100 in B16 cells (Figure 2C). The results showed that, in parallel with apoptosis, SR-T100 induced a dose-dependent increase in DNA damage. Significant DNA damages (>80%) were also observed after treatment with SR-T100 at 2 μg/mL for 24 hours.

### Caspase 9 mediates SR-T100-induced DNA damage and apoptosis

**Figure 3:**
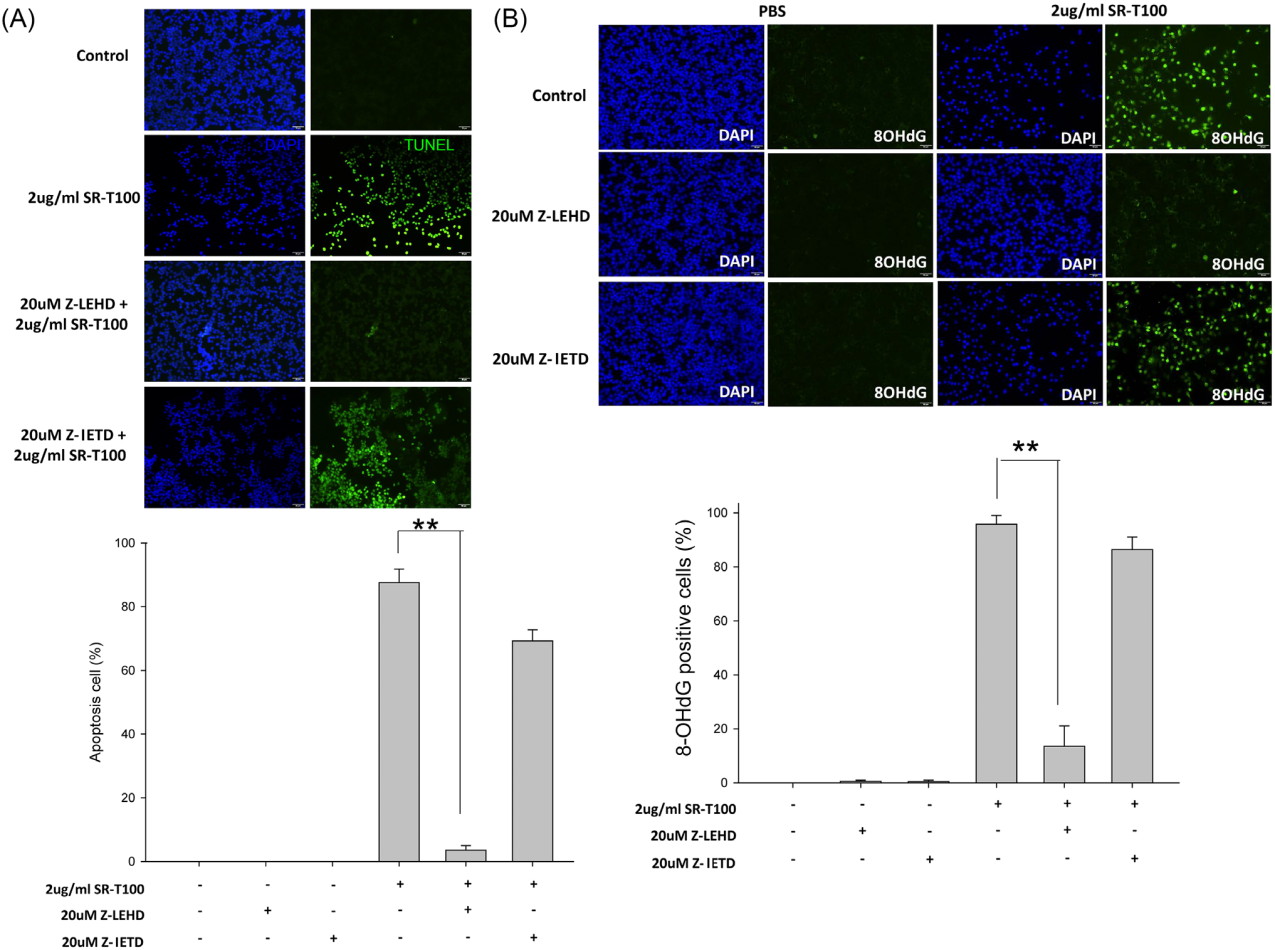
Caspase 9, but not caspase 8, mediates cell apoptosis and DNA damage in B16 cells induced by SR-T100 B16 cells were pretreated with z-LEHD (caspase 9 inhibitor) or z-IETD (caspase 8 inhibitor) at 20 μM for 2 hours before treatment of SR-T100 at 2 μg/mL for 24 hours. Cells were processed for immunofluorescence examination for TUNEL **(A)** or 8-OHdG **(B)**. Data from a representative experiment is shown in the photographs. The percentage of TUNEL staining cells or 8-OHdG stained cells was determined. Experiments were repeated three times in triplicate. ^*^ indicates p < 0.05, ^**^ indicates p < 0.01.

To address whether cell apoptosis and DNA damage were caspase-9 or -8 dependent, B16 cells were pre-treated with the caspase-9 inhibitor (20 μM Z-LEHD-FMK) or caspase-8 inhibitor (20 μM Z-IETD-FMK) for 2 hours before SR-T100 treatment. Cell apoptosis and DNA damage were measured by TUNEL assay (Figure 3A) and 8-OHdG expression (Figure 3B), respectively. The results showed that both apoptosis and DNA damage were rescued by pre-treatment with Z-LEHD-FMK, but not by pre-treatment with Z-IEDH-FMK. Collectively, the results indicated that caspase 9, but not caspase 8, mediates cell apoptosis and DNA damage induced by SR-T100.

### SR-T100 inhibits lung metastasis of melanoma *in vivo*

**Figure 4:**
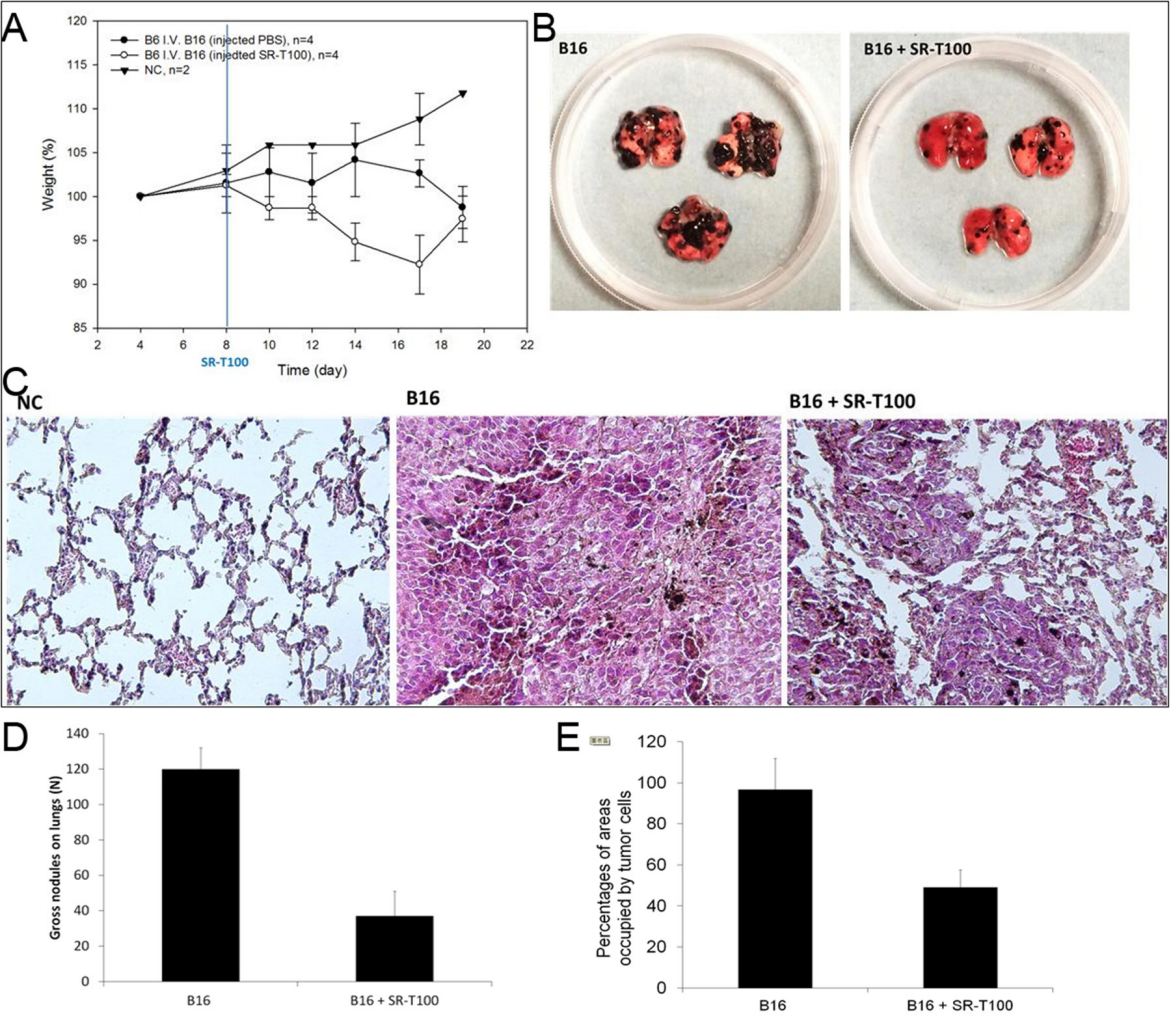
Effect of SR-T100 on metastasis *in vivo* **(A)** SR-T100 reduced the tumor burden of established lung metastasis in C57BL/6 mice. Lung metastases were established by injection of B16 cells via the tail vein. SR-T100 (B16 + SR-T100 group) or PBS (B16 group) were injected intraperitoneally daily from day 8 to day 18. **(B)** At day 19, mice were euthanized and the lungs were inspected for tumor burden. Representative data from three experiments are shown. **(C)** Histopathology showed SR-T100 reduced cell numbers and micro-nodules of metastatic melanoma in the lungs (H&E, 100X). **(D)** The numbers of gross nodules on the lungs from (B) were averaged and compared (three experiments). **(E)** The percentages of area occupied by the tumor cells were averaged from five power fields (100×) from (C).

Since we demonstrated the apoptotic effects of SR-T100 on B16 *in vitro*, we next determined whether SR-T100 would preferentially kill and induce adverse effects in an experimental lung metastasis model using B16 cells *in vivo*. B16 cells were injected intravenously into C57BL/6 mice to allow the establishment of lung metastasis by B16 cells. After 7 days, 4 mice were treated with intraperitoneal injection of 25 μL SR-T100 (5 mg/mL) and 4 other mice used as controls were treated with intraperitoneal injection of 25 μL PBS every day for 11 days. At day 19, mice were euthanized and lungs were inspected for tumor burden. Compared to control mice, mice that received B16 cell injection showed body weight loss (Figure 4A). For SR-T100-treated mice, the tumor burden of lung metastases was significantly reduced compared to that in control mice (Figure 4B and 4D). Histopathology using hematoxylin and eosin staining (H&E) showed that mice treated with SR-T100 had fewer microscopic nodules of metastatic melanoma in the lungs (Figure 4C and 4E). The Melan-A immunofluorescence staining on the lung tissues from three mice in each group validated the results obtained from regular H&E staining (Supplementary Figure 1). Together, these results showed that intravenous injection of SR-T100 inhibited established lung metastasis of melanoma *in vivo*.

### Intralesional injection of SR-T100 inhibits the growth of localized melanoma

**Figure 5:**
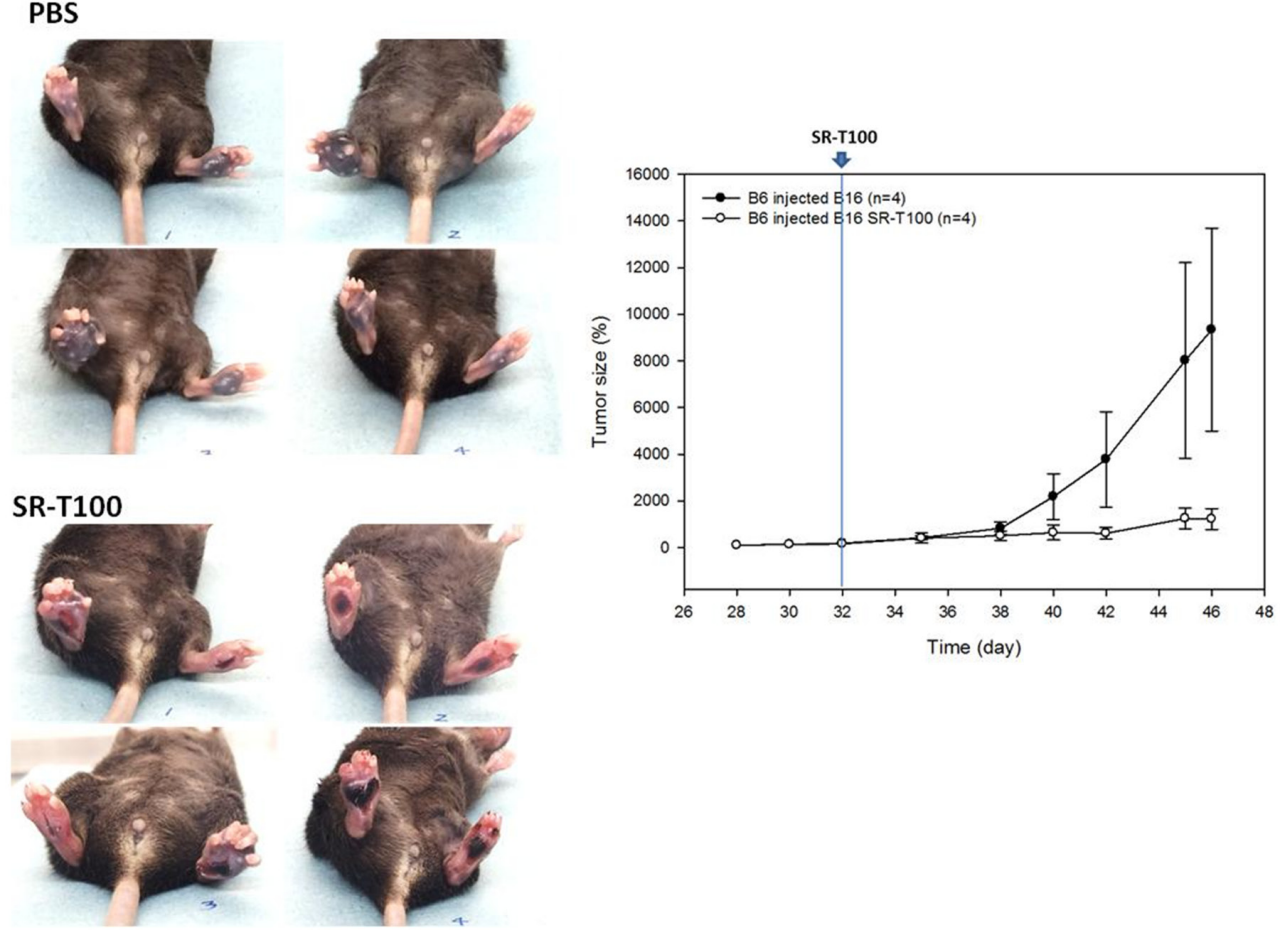
SR-T100 reduces the tumor size of localized melanoma in C57BL/6 mice Localized melanoma was established by injection of B16 cells into the footpads.SR-T100 or PBS was injected into the melanoma daily since day 32. Tumor volume was estimated as the product from length, width, and height. Representative data from three experiments are shown (n = 4 each group).

To further evaluate whether SR-T100 inhibited the growth of localized melanoma, we first established the local tumor by injection of B16 cells in the footpad. After melanoma was established in the footpads, 20 μL of SR-T100 (5 mg/mL) was then intralesionally injected to treat the regional melanoma (Figure 5). The results showed that tumor size was significantly lower and necrosis was higher in mice treated with intralesional injection of SR-T100 than in control mice, indicating that SR-T100 has a therapeutic effect against localized melanoma when introduced directly into the tumor.

## DISCUSSION

This study is the first to report that SR-T100 inhibits melanoma cells *in vitro* and reduces the growth of established metastatic melanoma *in vivo*. SR-T100 induced apoptosis and caused a G0/G1 cell cycle arrest in melanoma cells. In mice, intravenous injection of SR-T100 reduced tumor load of metastatic melanoma in the lung and intralesional injection of SR-T100 inhibited the growth of primary melanoma in the footpad. These results highlight SR-T100 as a potential therapeutic agent for metastatic melanoma.

In this study, we showed that SR-T100 induced apoptosis and caused G0/G1 cell cycle arrest in melanoma cells. Several mechanisms can explain how SR-T100 inhibits cancer growth. Several studies showed that SR-T100 and its active ingredient, solamargine, when used alone, can induce apoptosis of cancer cells [7, 8, 17]. Furthermore, solamargine can down-regulate HER2/neu receptors and enhance susceptibility of breast cancer cells to chemotherapy and immunotherapy [10–12]. Other mechanisms related to anticancer effects of SR-T100 include down-regulation of aldehyde dehydrogenase 1, which is a marker of cancer stem-like cells [12]. Because of the partial effects of SR-T100 in reducing melanoma growth and metastasis in mice, understanding whether SR-T100 can have a synergic effect with current therapies used against melanoma, including target therapy and immunotherapy, and if other mechanistic cascades are involved in the anti-melanoma effect of SR-T100 will be essential. Indeed, combined immunotherapy has shown higher efficacy against melanoma than monotherapy [4, 18]. Further investigations are warranted to evaluate the potential role of SR-T100 as an adjuvant therapy together with target therapy or immunotherapy.

Intralesional treatments for unresectable melanoma have garnered the attention in recent years. For instance, intralesional injection of talimogene laherparepvec, an oncolytic virus, has been approved for the treatment of melanoma lesions in the skin and lymph nodes in the United States [19]. Moreover, intralesional treatment has also been explored as a neoadjuvant treatment for resectable melanoma [20]. Notably, our experiments showed that intradermal injection of SR-T100 caused melanoma shrinkage in localized established melanoma. Further investigations are warranted to evaluate SR-T100 as a new intralesional treatment for unresectable melanoma and a neoadjuvant therapy for resectable melanoma.

In this study, SR-T100-treated mice lost more weight than control mice. In fact, in preclinical experiments performed for the development of our US patent, a 13-week subchronic oral toxicity study was performed. Animals significantly (*p* < 0.05) lost weight from the third week to the 13^rd^ week in the high dose group (2000 mg/kg/day), and from the 8^th^ week to the 13^rd^ week in the middle dose group (1000 mg/kg/day). Microscopically, SR-T100 induced basal vacuolation, ulceration, and necrosis of the forestomach (Supplementary Figure 2) as well as cysts in the submucosa of the forestomach, and hyperplasia and hypertrophy of the duodenum mucosal epithelium (data not shown) in a dose-dependent manner. Thus, changes in body weight are associated with injuries in the gastrointestinal mucosa induced by SR-T100 treatment. This might explain why mice treated with SR-T100 lost weight.

In conclusion, this study showed that SR-T100 induces melanoma cell apoptosis *in vitro* and inhibits metastatic melanoma and localized melanoma *in vivo*. Our research highlights SR-T100 as a potential novel therapeutic for melanoma. Although induction of apoptosis can account for the anticancer effect, it is worth investigating whether other mechanisms such as anti-angiogenesis and immune stimulation are responsible for the anticancer effect of SR-T100 against melanoma. Animal studies with larger sample size are warranted to validate the efficacy and the optimal dosage of SR-T100.

## MATERIALS AND METHODS

### Preparation of *Solanum incanum* extract

*Solanum incanum* extract was manufactured from *Solanum incanum* (synonym: *Solanum undatum*) according to the patents (US patent 7,078,063, EU patent 1,058,334, and Japan patent 3,940,928). Briefly, the active component, solamargine, was extracted by acid-base precipitation followed by the different ratios of ethanol/H_2_O extraction. The final extract was then dried by lyophilization. Solamargine in *Solanum incanum* extract was quantified by reverse-phase high performance liquid chromatography and diluted to a concentration of 10 mg/mL with pure water as a stock solution. The chromatographic fingerprint of *Solanum undatum* extract revealed two major components, solamargine and solasonine as identified by comparisons with solamargine and solasonine standards (APIN Chemicals, UK). *Solanum incanum* extract contains solamargine and solasonine with the ratio approximately 62 to 38, and less than 1% of hydrophilic minor components. Our previous studies demonstrated that solamargine was the major active component of *Solanum incanum* against a SCC cell line (SCC25). The cytotoxic activity of solasonine was 17 times lower than that of solamargine. Furthermore, other hydrophilic minor components within *Solanum incanum* extract did not show cytotoxicity to tumor cells [17]. Thus, solamargine served as a standard in the present study for quality control. Since SR-T100, solamargine in *Solanum incanum* extract, is currently evaluated in clinical trials under US and Taiwan FDA approvals, the analytical method for quantifying solamargine is validated according to the standard pharmaceutical procedures, including GMP.

### Cell culture

Murine B16 melanoma cells, human A375 melanoma cells, and human G361 melanoma cells were maintained in Dulbecco’s minimal essential medium (DMEM) supplemented with 10% fetal calf serum (FCS) and 100 U/mL penicillin G and 100 μg/mL streptomycin sulfate (Gibco, Thermo Fisher Scientific, Waltham, MA, USA). Cells were passaged at confluence after treatment with 5mM EDTA (Gibco, Thermo Fisher Scientific) and incubated at 37°C in a humidified atmosphere containing 5% CO_2_.

### Cell viability

Cell viability was assessed by the Cell Counting Kit-8 (CCK-8) assay (Sigma-Aldrich, St. Louis, MO, USA). Briefly, cells were seeded in a 96-well plate at the cell density of 5000 cells/well. After 24 h pre-incubation in a humidified incubator (37°C, 5% CO2), each well was incubated with 10 μL CCK-8 for 4 h. Finally, the optical density was read at 450 nm with a microplate reader (MRX-II, Dynex technology, Chantilly, VA, USA).

### Cell cycle phase analysis

Cells were treated with compounds at 4 μM for 24 h. Cells were harvested by trypsinization and centrifugation. Cell pellets were resuspended in 70% cold ethanol and fixed for 30 min at 4°C. After fixation, cells were washed twice with cold PBS and centrifuged at 850 × *g*. The supernatant was carefully discarded to avoid cell loss. The cells were treated with 50 μL of a 100 μg/mL RNase solution and 200 μL of propidium iodide (50 μg/mL stock solution). Thirty minutes later, the DNA content of 10,000 events was measured by FACScan flow cytometer (Elite ESP, Beckman Coulter, Brea, CA, USA). Histograms were analyzed by using Windows Multiple Document Interface software (WinMDI).

### TUNEL assay

To detect apoptotic cells, a TUNEL kit (Roche, Mannheim, Germany) was used. Tissue slides were washed twice with PBS, and the slides were incubated in permeabilization solution (0.1% Triton X-100 in 0.1% sodium citrate) for 2 min on ice. Permeabilized tissue sections were washed with PBS. After washing, 50 μL of TUNEL reaction mixture was added to each sample. A 60 min reaction at 37°C was required to label the DNA strand breaks with PE. Percentages of TUNEL positive cells were analyzed under a fluorescence microscope (BX53, Olympus, Tokyo, Japan).

### 8-hydroxy-2’-deoxyguanosine (8-OHdG) DNA damage assay

Cells were fixed with 4% paraformaldehyde in PBS for 10 min at room temperature. The cells were incubated for 10 min with PBS containing 0.25% Triton X-100 at room temperature. After cells were washed in PBS three times for 5 min, the cells were incubated in 5% BSA in PBS for 1 h at room temperature. Cells were then incubated with 8-OHdG-FITC antibody at 1:50 dilution (sc-393871, Santa Cruz Biotechnology, Santa Cruz, CA, USA) in 5% BSA in PBS overnight at 4°C before examination using a fluorescence microscope. For caspase blocking, cells were pretreated with caspase-9 inhibitor (BD bioscience 550381, Z-LEHD-FMK, BD Biosciences, San Jose, CA, USA) or caspase-8 inhibitor (BD bioscience 550380, Z-IEDH-FMK) for 2 hours before treatment with SR-T100.

### Animals

Male C57BL/6 mice were obtained from National Laboratory Animal Center in Tainan, Taiwan. All mice were housed in a specific pathogen-free animal facility. Experimental procedures were approved by the Animal Care and Use Committee of the Kaohsiung Chang Gung Memorial Hospital. Lung metastases were established by injection of B16 cells (400 μL, cell density: 2.2 × 10^6^ cells/mL) via tail vein. B16 cells in exponential growth phase were harvested by trypsinization and washed twice with PBS before injection. Intraperitoneal injection of 25 μL SR-T100 (5 mg/mL) was administered into 4 mice daily from day 8 to day 18, while 4 control mice were intraperitoneally injected with PBS from day 8 to day 18. At day 19, mice were euthanized and the lungs were harvested to inspect melanoma tumor burden. Tissues from the lungs were processed for routine formalin fixation, paraffin embedding, sectioning, and H&E staining as well as immunofluorescence staining for DAPI and Melan-A (1:1000, Abcam), a specific marker for melanocyte-lineage cells. To evaluate the efficacy of intralesional injection of SR-T100 against established melanoma, B16 melanoma cells (30 μL, cell density: 2.2 × 10^6^ cells/mL) were injected into the mouse footpads. Four mice received 20 μL intralesional injection of SR-T100 (5 mg/mL) and four mice received intralesional injection of PBS since day 32. At day 46, mice were euthanized and tumor sizes were measured.

### Statistical analysis

The numeric variables among two groups (for example, the tumor size between the treated group and control group at a given time point, the densitometric data from western blot, the mean fluorescence intensity in TUNEL assay) were compared by Student’s *t*-test. The ratio variables (for example, the ratio of cell in each cell cycle phase) were compared by Chi-square test. The numeric variables among several groups were compared by ANOVA with post-comparison Scheffe’s test. The statistical analysis was performed by using SPSS ver 14 (Chicago, IL, USA). A p-value less than 0.05 was considered significant.

## SUPPLEMENTARY MATERIALS FIGURES



## References

[1] Ekwueme DU, Guy GP, Li C, Rim SH, Parelkar P, Chen SC (2011). The health burden and economic costs of cutaneous melanoma mortality by race/ethnicity-United States, 2000 to 2006. J Am Acad Dermatol.

[2] Balch CM, Buzaid AC, Soong SJ, Atkins MB, Cascinelli N, Coit DG, Fleming ID, Gershenwald JE, Houghton A, Kirkwood JM, McMasters KM, Mihm MF, Morton DL (2001). Final version of the American Joint Committee on Cancer staging system for cutaneous melanoma. J Clin Oncol.

[3] McArthur GA, Chapman PB, Robert C, Larkin J, Haanen JB, Dummer R, Ribas A, Hogg D, Hamid O, Ascierto PA, Garbe C, Testori A, Maio M (2014). Safety and efficacy of vemurafenib in BRAF(V600E) and BRAF(V600K) mutation-positive melanoma (BRIM-3): extended follow-up of a phase 3, randomised, open-label study. Lancet Oncol.

[4] Postow MA, Chesney J, Pavlick AC, Robert C, Grossmann K, McDermott D, Linette GP, Meyer N, Giguere JK, Agarwala SS, Shaheen M, Ernstoff MS, Minor D (2015). Nivolumab and ipilimumab versus ipilimumab in untreated melanoma. N Engl J Med.

[5] Kupchan SM, Barboutis SJ, Knox JR, Cam CA (1965). Beta-solamarine: tumor inhibitor isolated from Solanum dulcamara. Science.

[6] Cham BE, Meares HM (1987). Glycoalkaloids from Solanum sodomaeum are effective in the treatment of skin cancers in man. Cancer Lett.

[7] Kuo KW, Hsu SH, Li YP, Lin WL, Liu LF, Chang LC, Lin CC, Lin CN, Sheu HM (2000). Anticancer activity evaluation of the solanum glycoalkaloid solamargine. Triggering apoptosis in human hepatoma cells. Biochem Pharmacol.

[8] Liu LF, Liang CH, Shiu LY, Lin WL, Lin CC, Kuo KW (2004). Action of solamargine on human lung cancer cells--enhancement of the susceptibility of cancer cells to TNFs. FEBS Lett.

[9] Shiu LY, Chang LC, Liang CH, Huang YS, Sheu HM, Kuo KW (2007). Solamargine induces apoptosis and sensitizes breast cancer cells to cisplatin. Food Chem Toxicol.

[10] Shiu LY, Liang CH, Huang YS, Sheu HM, Kuo KW (2008). Downregulation of HER2/neu receptor by solamargine enhances anticancer drug-mediated cytotoxicity in breast cancer cells with high-expressing HER2/neu. Cell Biol Toxicol.

[11] Shiu LY, Liang CH, Chang LC, Sheu HM, Tsai EM, Kuo KW (2009). Solamargine induces apoptosis and enhances susceptibility to trastuzumab and epirubicin in breast cancer cells with low or high expression levels of HER2/neu. Biosci Rep.

[12] Wu YH, Chiu WT, Young MJ, Chang TH, Huang YF, Chou CY (2015). Solanum incanum extract downregulates aldehyde dehydrogenase 1-mediated stemness and inhibits tumor formation in ovarian cancer cells. J Cancer.

[13] Cham BE, Daunter B (1990). Solasodine glycosides. Selective cytotoxicity for cancer cells and inhibition of cytotoxicity by rhamnose in mice with sarcoma 180. Cancer Lett.

[14] Liang CH, Shiu LY, Chang LC, Sheu HM, Kuo KW (2007). Solamargine upregulation of Fas, downregulation of HER2, and enhancement of cytotoxicity using epirubicin in NSCLC cells. Mol Nutr Food Res.

[15] Hsu SH, Tsai TR, Lin CN, Yen MH, Kuo KW (1996). Solamargine purified from Solanum incanum Chinese herb triggers gene expression of human TNFR I which may lead to cell apoptosis. Biochem Biophys Res Commun.

[16] Chang LC, Tsai TR, Wang JJ, Lin CN, Kuo KW (1998). The rhamnose moiety of solamargine plays a crucial role in triggering cell death by apoptosis. Biochem Biophys Res Commun.

[17] Wu CH, Liang CH, Shiu LY, Chang LC, Lin TS, Lan CC, Tsai JC, Wong TW, Wei KJ, Lin TK, Chang NS, Sheu HM (2011). Solanum incanum extract (SR-T100) induces human cutaneous squamous cell carcinoma apoptosis through modulating tumor necrosis factor receptor signaling pathway. J Dermatol Sci.

[18] Larkin J, Chiarion-Sileni V, Gonzalez R, Grob JJ, Cowey CL, Lao CD, Schadendorf D, Dummer R, Smylie M, Rutkowski P, Ferrucci PF, Hill A, Wagstaff J (2015). Combined nivolumab and ipilimumab or monotherapy in untreated melanoma. N Engl J Med.

[19] Greig SL (2016). Talimogene laherparepvec: First global approval. Drugs.

[20] Weide B, Neri D, Elia G (2017). Intralesional treatment of metastatic melanoma: a review of therapeutic options. Cancer Immunol Immunother.

