# Supplementary material for: Solanum incanum extract (SR-T100) induces melanoma cell apoptosis and inhibits established lung metastasis
Source: Oncotarget. 2017 Oct 4;8(61):103509–17. doi: 10.18632/oncotarget.21508 (PMC5732746; doi:10.18632/oncotarget.21508)
Supplement: Supplementary file 1 [file oncotarget-08-103509-s001.pdf]

Solanum incanum extract (SR-T100) induces melanoma cell apoptosis and inhibits established lung metastasis

SUPPLEMENTARY MATERIALS

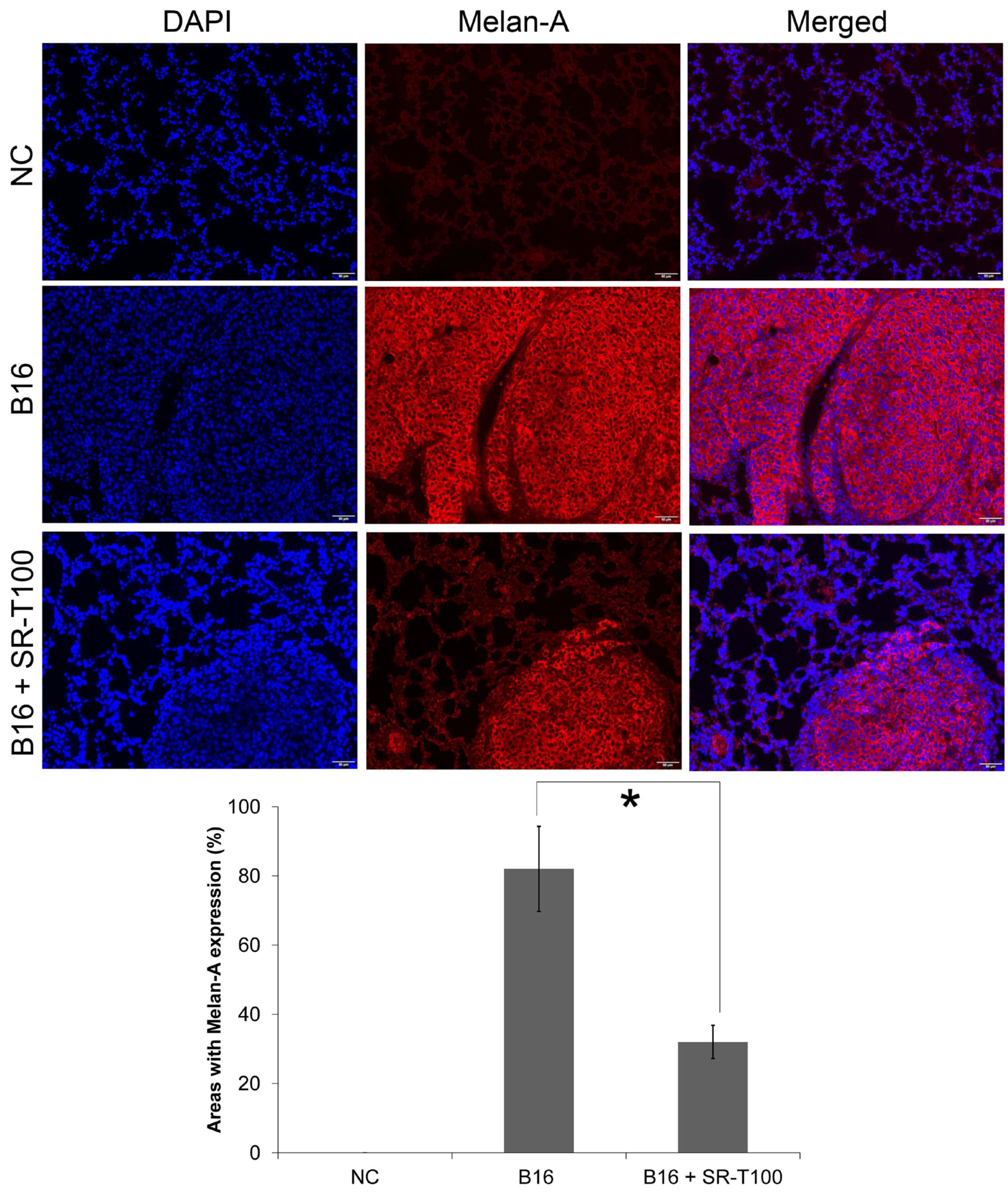

Supplementary Figure 1: Lung metastases were established by injection of B16 cells via the tail vein. SR-T100 (B16 + SR-T100 group) or PBS (B16 group) were injected intraperitoneally daily from day 8 to day 18. At day 19, mice were euthanized and the lungs were inspected for tumor burden and stained for Melan-A (100×). Representative data from three experiments are shown. The percentages of area expressing Melan-A were averaged from five power fields for comparisons among three groups. * denotes $p < 0.05$.

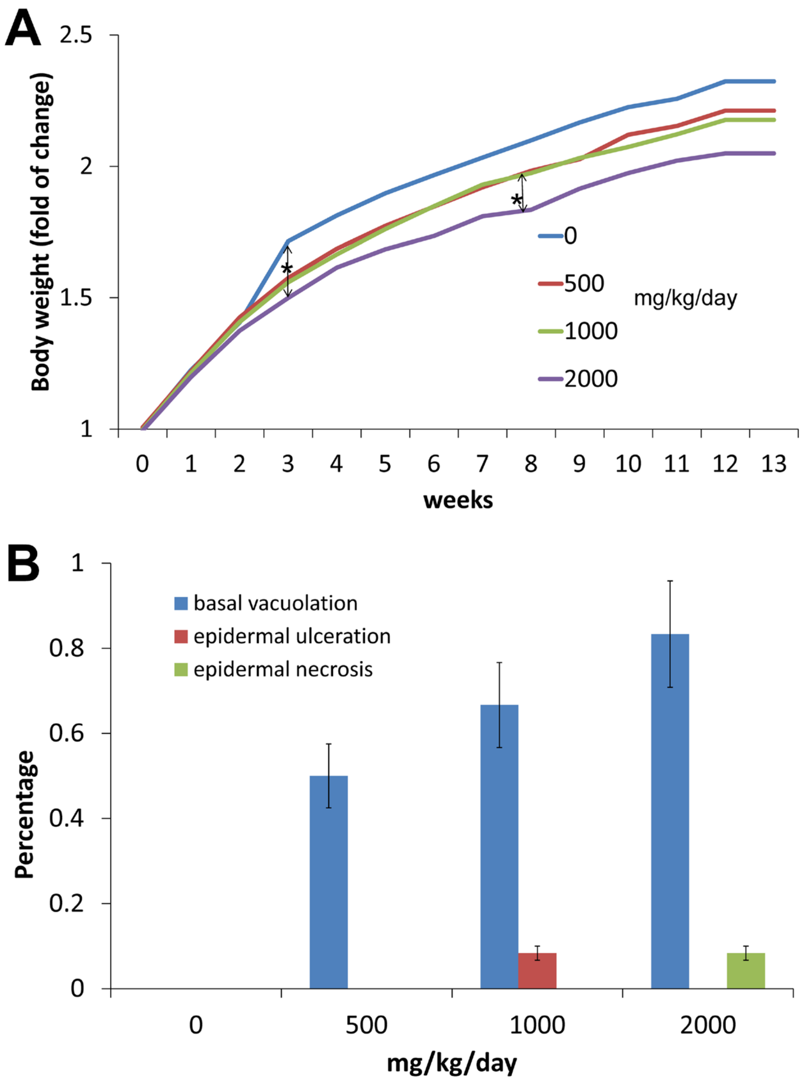

Supplementary Figure 2: SR-T100 cytotoxicity. **(A)** Animals significantly lost weight from the third week to the 13rd week in the high dose group (2000 mg/kg/day), and from the 8th week to the 13rd week in the middle dose group (1000 mg/kg/day). n = 12 for each group, * indicates $p < 0.05$ at that time and afterwards. **(B)** Microscopically, SR-T100 induced basal vacuolation, ulceration, and necrosis of the forestomach in a dose-dependent manner.
